# Theory of Motivated Cue-Integration and COVID-19: Between Interoception, Somatization, and Radicalization

**DOI:** 10.3389/fpsyt.2021.631758

**Published:** 2021-06-11

**Authors:** Idit Shalev

**Affiliations:** Laboratory for Embodiment and Self-Regulation, Department of Psychology, Ariel University, Ariel, Israel

**Keywords:** COVID-19, motivated cue-integration, radicalization, somatization, interoception, embodied cognition, self-regulation, self-regulation failure

## Abstract

The global dissemination of COVID-19 creates confusion and ambiguity in nearly every aspect of life, including fear of contagion, heightened awareness of the mortality of self and family members, lack of power, and distrust of experts and decision-makers. In this stressful situation, the question arises as to what mechanisms distinguish between adaptive and maladaptive self-regulation. The theory of Motivated Cue-Integration (MCI) is a novel theory of self-regulation that provides a new perspective on the effect of COVID-19 on self-regulation deficiency as an example of psychological distress. Inspired by predictive coding, social cognition, embodied cognition, and experiential approach, MCI suggests that self-regulation is based on interaction between (1) high-level values and goals, (2) low-level interoceptive and exteroceptive signals, and (3) trust in epistemic authority or a significant other. Motivated Cue-Integration posits that individuals create meaning by making moment-to-moment predictions that affect their interpretation of the experience of ambiguity influenced by their relationship with epistemic authority. According to MCI, deficiency in self-regulation during COVID-19 could result either from over-sensitivity or under-sensitivity to low-level interoceptive and exteroceptive cues; rigidity or ambiguity of high-level goals, poor integration between the two levels of processing as well as distrust in epistemic authority. According to MCI, variations of these deficiencies may occur in various clinical phenomena such as alexithymia and somatization, as well as in social phenomena such as goal radicalization. Based on this reasoning, MCI claims that the mentalization of the relationship between interoceptive cues, exteroceptive cues, goals, and psychological needs of the person, as well as the improvement of confidence in epistemic authority, can promote adaptive self-regulation. Psychological intervention can foster trust in epistemic authority, increase the mentalization of interoceptive and exteroceptive cues, and their association with adaptive goals. As such, the integration of these elements in a way that facilitates incentives pathways and insight fosters a more integrated subjective experience, higher clarity of emotion, and positive internal dialogue which promotes action tendency.

## Introduction

The global spread of COVID-19 due to severe acute respiratory syndrome (SARS-CoV-2) has generated uncertainty and ambiguity in almost every aspect of life. According to the World Health Organization as of mid-March 2021, more than 120 million cases have been confirmed, with more than 2.66 million deaths attributed to COVID-19, making it one of the deadliest pandemics in history. For many people, COVID-19 has created fear of contagion, increased awareness of the death of self and family members, economic crisis, loss of autonomy as well as distrust of experts and policy makers. The psychological effects of quarantine have been associated with post-traumatic stress, uncertainty, anger, fear of infection, dissatisfaction, boredom, inadequate supplies, and unreliable information (1). Despite these conditions, at the same time, some individuals have been engaged in cooperative and altruistic behavior and provided social and emotional support (2). In this stressful condition, the question arises as to what mechanisms distinguish between adaptive and maladaptive self-regulation. Theories of self-regulation have mostly related to control processes (3), and very little is known about the role of interoception and exteroception in the creation of subjective meaning and self-regulation, particularly in the context of pandemic. Based on the theory of Motivated Cue-Integration (MCI) (4–7), this paper will discuss the nature of adaptive vs. maladaptive self-regulation in the context of the COVID-19 pandemic.

In what follows, I will first present the novel theory of MCI as compared to traditional self-regulation and its association with predictive coding. Next, I will describe how pandemic-based insecurity affects the three pillars of MCI. Finally, I will address psychological guidelines that can improve the process of motivated integration of cues in a state of uncertainty.

## What Is Self-Regulation?

Self-regulation acts through a negative feedback loop which monitors the individual's current state against a reference value. Once a discrepancy is found, the person interacts with the current state and the desired state to minimize the discrepancy. In the context of health, self-regulation is seen as a personal resource that enables an individual to engage in objective behaviors that affect health, or as a set of behaviors, or as skills that can be learned and put into practice to promote health (8). At the core of self-regulation is the goal concept defined as cognitive representation of a desired end state that affects evaluations, emotions, and behavior (9). Goals include information on the desired states, which serves as a reference point to which behavior is directed (10–12). Individual life goals represent one's attempts to accomplish personal self-change, as well as enhance meaning and purpose in life (13). Accordingly, adaptive self-regulation requires selection and implementation of appropriate means to attain goals (4, 10, 11). Self-regulation can automatically be activated by contextual cues (e.g., word, image, metaphor, sound, smell), which activate goal representation and, subsequently, influence judgment and behavior without conscious awareness (14, 15). The automatic aspect of self-regulation is related to habits and does not require attention resources. Self-regulation involves cognitive and motivational properties. The cognitive properties are related to executive function such as focus of attention and working memory. The motivational properties include action selection, effort valuation, performance, reward learning, and reward expectations. Accordingly, self-regulation failure results in various deficits in these functions (5).

## What Are the Traditional Theories of Self-Regulation Lacking?

Self-regulation theories describe numerous psychological phenomena related to action initiation, decision-making, self-control, and impulse control (16). However, some self-regulation processes have yet to be studied. First, while the psychology of action may be analogously linked to a car driven by both its energy and its goal directed action (17), research has been more concerned with self-regulation of goals than with regulation of energy. Second, based on dual process theories involving automatic vs. deliberated processes (18), self-regulation has been primarily concerned with goal-setting and goal-striving processes (19), and less is known about the effect of awareness of sensation on recognition of internal needs or personal goals, especially in the context of ambivalence and uncertainty. Third, research on self-regulation was primarily associated with a mechanistic cognitive approach or judgment of external reality, while the experiential aspect was underestimated. Fourth, self-regulation models suggest that goals are activated by exteroceptive contextual cues (20), and less is known about the effect of interoceptive cues on self-regulation (6, 7). Last, self-regulation theories have mainly focused on the “Self” and less is known on relational aspects of self-regulation. To address these gaps, the theory of MCI (4–6) proposes a new look at self-regulation, which integrates a predictive coding model, social cognition, embodied cognition research as well as a phenomenological approach. In what follows, I will first describe the predictive coding model. I will then present the fundamental assumptions of MCI in relation to deficiencies in self-regulation and relate MCI applications to COVID-19. Finally, I will include guidance for the course of therapeutic action resulting from MCI.

## Predictive-Coding

According to predictive coding, the brain proactively adapts the body's physiological systems to meet needs before they arise. The brain has to find information about the potential explanations for sensory indications (i.e., perception) without direct exposure to these sources (21), for bodily navigations in the environment and reducing free-energy efficiency in internal states (22). This process is also influenced by the need to minimize the cost of prediction error, either by updating generative models or by taking action to link sensory states in line with predictions (23). Basically, the brain is asking what input is most similar to where similarity is calculated against population predictions and their associated costs and the potential benefits of the product. These predictions are derived from Bayesian brain inferencing and all are about energy balance (24, 25). The process is promoted by the presence of two types of inputs: (1) exteroceptive inputs associated with body perception from the outside, based on multisensory integration, and (2) interoceptive inputs (26), classified as the sense of the inner physiological state that promotes homeostatic regulation of the body, culminating in physiological integrity and associated affective states, drives, and emotions (27, 28). The traditional predictive coding model suggests that the difference between internal state and environmental prediction is assessed by Bayesian statistics and described as a “surprise” that minimizes continuous system repairs. The system can also minimize the gap by intentionally changing the environment to the expected state. Prediction error is unanticipated information that comes from both internal and external sensory domains and modulates predictions. Error signals that track the difference between the sensations predicted and those coming from the sensory world are called precision signals. These signals calculate the prediction error from the incoming sensory input and optimize the sampling of the sensory periphery for allostasis. Unexpected sensory inputs that are expected to have allostatic implications because they are likely to have an impact on survival, reward or threat, or are of uncertain value, will be treated as “signal” and learned (i.e., encoded) to better predict energy needs in the future, with all other prediction errors treated as “noise” and safely ignored.

The theory of MCI (4–6) is a novel theory of self-regulation, indicating the existence of analogous mechanisms of brain and mental processes and integrating an experimental research perspective of social cognition and embodied cognition along with an experiential approach.

## Theory of Motivated Cue-Integration

The theory of MCI (4–6) suggests that the need for moment-to moment prediction is at the core of self-regulation. According to MCI, self-regulation is activated by both bottom-up and top-down processes through selective attention to goals and psychological needs, multisensory information, contextual cues, and affective signals, which, in turn, are integrated into meaning, resulting in action generation (4).

Following the perspective of “motivation as cognition” that attributed different functions to motivational and cognitive variables, suggesting that motivation will fluctuate between one moment to the next, thereby defining the degree to which any type of knowledge (strategic and peripheral; conscious and unconscious) is interpreted (29), MCI posits that not only do motivations fluctuate between one moment to the next but also interoceptive and exteroceptive cues are all integrated with high-level goals (4). Accordingly, the link between control processes and incentives through the term “goal” enables an understanding of the relationship between distinct dorsolateral and ventromedial brain-separated systems (6). Likewise, MCI fills an additional gap in the literature of embodied cognition. According to this school of thought, higher-level processing is based on the lower level sensory and motor experiences of the organism (30–33), indicating that activation automatically spreads from concepts based on experience in the physical world to their metaphorically associated social concepts [for reviews, see (32, 34)]. However, the study of perceptual symbols yielded various patterns of activation, which thus render assumptions about specific judgment and behavior problematic (4). Based on the shift in cognitive science that cognitive processes and their underlying neuronal activity patterns should be investigated primarily with respect to their role in generating action (35), Shalev (4) proposed that embodied cues are integrated according to their momentary functions within each individual's system of goals.

Following this view, MCI suggests that individuals consistently construe meaning based on relations between three pillars enabling self-regulation: (1) the low-level homeostatic moment-by-moment aspect of self-regulation which takes place through attention to emotion, interoceptive, and exteroceptive cues; (2) the high-level aspect of self-regulation, associated with individual goals, values, and aspirations; and (3) the continuous relationship of trust vs. distrust with epistemic authority (e.g., significant other, government, religious authority) which aims to reduce ambiguity, as elaborated in the next section.

## The Three Pillars of Motivated Cue-Integration

### High Level Processes

High level processes relate to individual goals, personal needs, values, and aspirations. A systemic view of human behavior and its implications for the course of action indicates that goal systems are a mentally represented network in which goals with appropriate means and alternative goals can be cognitively associated (11). Whereas, the classic goal systems theory (11) is mostly related to general processes, MCI is focused on individual differences. The individual differences in perception of the social context are conveyed by previous patterns that created unique associations between goals and means of attainment (4), repeated coupling of sensory signals (36), and strength of the association between particular physical sensations and psychological concepts such as the combination of homeostatic cues (e.g., temperature and dryness). In addition, situational demands, history, and psychiatric and neuropsychological conditions (e.g., cognitive flexibility) influence MCI (4, 37). Ample research has been carried out on the relationship between the structure and function of the individual's goal system (38, 39), including the substitution of means, conflicting goals (12), rigidity, and radicalization of goals resulting in self-destruction (40, 41). The motivational relevance of the individual indicates the degree and duration of the goals of the individual in the present context thus influence its valence in cue-integration, resulting in adaptive vs. maladaptive self-regulation. Value relevance, for example, refers to the extent to which acts of mental representation produce the desired results or prevent unwanted outcomes; control relevance relates to the efficacy with which active representation produces things; and truth relevance evaluates what is real (42).

### Low Level Processes

Earlier emotion theories demonstrated the difference between high-level and low-level processes (43–45). For example, James and Dennis' (43) psychological theory related visceral-afferent input and emotional experience. Whereas, research on automatic self-regulation indicates that contextual cues (e.g., word, image, metaphor, sound, smell) automatically activate associated goal representations that subsequently influence judgment and behavior (14, 15), MCI emphasizes the importance of both interoceptive and exteroceptive signals (46, 47). Research on embodied cognition suggests that contextual and interoceptive visceral cues (e.g., temperature, dryness) carry contextual meaning, as well as cultural or idiosyncratic interpretation (47–50).

Likewise, whereas the traditional predictive coding model suggests that the difference between internal state and environmental prediction is assessed by Bayesian statistics, MCI relates to the motivated aspect of cue-integration, including subjective cognitive distortions as well as cultural influences. For example, Shalev (46) provided evidence that exposure to contextual cues associated with dryness and experience of physical thirst created by salty food results in procrastination of decision making and lower persistency in unsolvable anagram tasks. The same cues influenced the subjective experience of fatigue, indicating the association between interoceptive and exteroceptive cues and their influence on the cost of action. Bodily cues are mentalized, evolve into emotions, and reflect a contextual assessment that influences judgment and behavior, as in the case of disgust that has evolved in human cultures to respond to immoral acts or low-level people (51).

### Trust in Epistemic Authority

The term epistemic authority refers to significant others, public leaders, policy makers, or God, suggesting individuals are willing to rely and accept information from epistemic authority as evidence of the truthfulness of the source's statements (29, 52). Motivated Cue-Integration claims that trust in epistemic authority reduces uncertainty. Ample research provides evidence of human dependence on external treatment in early life. This dependence enables advanced integration and organization of sensory and motor signals, resulting in the formation of a minimal self and brain mechanisms for incarnated mentalization (53). Evidence shows that attachment figures play a significant role in individual exploration and self-regulation (54–58). Following this view, Fonagy (59) suggests that epistemic trust in relationships is based on the person's experience of communicating with others, in particular the ability to receive and manage new information from others as relevant. Under ambiguity, the more individuals have confidence in the information provided by external sources, the more they can make autonomous predictions. On the other hand, the more distrust they have in the epistemic authority, the more dependence they have on external resources. Among adults, evidence shows that the more individuals trust the information provided by external sources (e.g., significant others, public leaders, policy makers), the more relatively autonomous predictions are made (60). According to expectancy-value models, this successful process increases both self-efficacy and hope (61).

## Deficits in Self-Regulation Based on the MCI Model

Inspired by Frijda (62), MCI suggests that specific emotions imply specific eliciting sensations, specific action tendencies, and specific differential reinforcement. When functioning appropriately, this process allows for adjusting to evolving environmental demands in a flexible manner. In comparison, inefficient affective information retrieval or a failure to associate embodied cues with top-down goals contribute to affective dysregulation and inaction. When this inefficiency becomes serious, various pathologies are shown to occur (63). Following this view, several sources influence deficits in self-regulation. First, rigidity or ambiguity of goals. Second, low level interoceptive and exteroceptive signals as well as from poor integration of the two. Accordingly, recent research demonstrates that both symptom similarities within psychological conditions and symptom heterogeneity between disorders may be dependent on interoception (64–66). The association between interoception and alexithymia illustrates symptom intercorrelations, suggesting that interoceptive capacity may be underlying the p-factor, a first-order overarching factor that defines the severity of psychopathology and its associated neural dysfunction and is revealed by conducting confirmatory factor analysis on the co-occurrence of specific symptoms across psychiatric diagnoses (67). Following this view, Paulus et al. (68) differentiated between two patterns of interoceptive dysfunction that resulted in interoceptive psychopathology. Firstly, people have abnormally high expectations of situations that cause changes in the body (i.e., hyper-precise priors) and, secondly, when the environment changes (i.e., rigidity of context), they have great difficulty adjusting these perceptions. Therefore, lack of ability to adjust expectations as a function of context can contribute to a constant experience of somatic error, as an individual in a new environment does not alter old assumptions about different models.

Furthermore, deficits in self-regulation could result from distrust in epistemic authority, especially under uncertainty (59, 69). Uncertainty can also manifest itself in a disconnect between goals, sensations, and emotions that leads to difficulties in evaluating goals and delays in action. The lack of certainty in interpreting the sensations of the body and the lack of validation of emotional experience reduces the sense of agency and increases the need for external control. In what follows, I will look at COVID-19 as a case study for self-regulation deficits based on the MCI model.

## COVID-19 as a Case Study for Self-Regulation Deficits Based on the MCI Model

### 1. Uncertainty and Distrust in Self and Epistemic Authority

Because pandemic management is extremely amorphous, individuals need information from external sources to integrate their experience and predict the future. The experience of certainty is defined as the conviction that the information available is real and trustworthy. A state of mind of lack of doubt or a sense of security (70) is central to adaptive self-regulation. Uncertainty is reflected in the lack of internal constraints between the interactive parts of the system, such that knowing the status of one component provides minimal information about the others (71). There is evidence that the higher the level of public trust in government, the more public policy support among residents will be observed (72, 73). Specifically, there is more pro-social behavior and self-sacrifice behavior among residents (74, 75). By contrast, more conspiracy theories were linked with lower adherence to COVID-19 preventive actions (76).

### 2. The Effect of COVID-19 on Low-Level Processes

#### Interoceptive and Exteroceptive Cue Misperception, Misinterpretation, and Miscommunication as Markers of Health and Disease

A fundamental experience of certainty comes into play through reality testing involving the orientation of time and place, the assumption that what is perceived exists, the experience of contrast between inner and outer realms, and a clear assessment of how individuals respond to their environment (77). According to MCI, the lack certainty in the interpretation of the body's sensations and the lack of validation of emotional experience impairs the sense of agency and increases the need for external control. Therefore, confusion and ambiguity about perception and interpretation of interoceptive and exteroceptive signals have been generated by COVID-19. For instance, while extensive research has emphasized the association between feelings of affection and trust in physical proximity, COVID-19 leads to the creation of an association between social proximity and insecurity that contradicts innate processes.

A fundamental experience of certainty comes into play through reality testing involving the orientation of time and place, the assumption that what is perceived exists, the experience of contrast between inner and outer realms, and a clear assessment of how individuals respond to their environment (77). The pandemic has created a lack of confidence in the sensation of the body as a health and disease marker, since without sensing or experiencing physical symptoms, one can be infected and infect others. Likewise, symptoms of impaired taste and smell damage the fundamental trust in body data as a source of prediction. Motivated Cue-Integration suggests that the lack of confidence in body signals as health or disease markers increases the reliance on external signals as data sources, as in the case of alexithymia (6, 7). While interoceptive and exteroceptive signals play a key role in human behavior, people's introspection of the causes of their own behavior and attitude has led to undervaluation of the impact of these low-level signals because they tend to interpret their own behavior as a result of conscious and rational decision-making (78).

#### Low Emotional Clarity, Alexithymia

Motivated Cue-Integration views emotional episodes as special types of goal directed action episodes (79), indicating that emotion is extended to external perception (i.e., contextualized: “I feel like that”) and transformed into a goal that comes into play through voluntary action (80). Following this view, Hommel et al. (79) argued that both emotional and non-emotional action trends are determined by high-level goal-directed processes, which differ only in the degree of control priority they have. Therefore, MCI suggests that greater clarity and awareness of emotion will result in action tendency. Emotional clarity deficits are linked to signs of depression, social anxiety, borderline personality disorder, binge feeding, and alcohol consumption, implying that emotional clarity deficits can be regarded as a transdiagnostic syndrome of divergent processes causing difficulties regulating emotions (81). These deficits are especially evident in alexithymia (82), which is described as a spectrum disorder characterized by difficulty identifying feelings and distinguishing them from bodily sensations of emotional arousal, difficulty describing one's own feelings, and an externally oriented cognitive style, i.e., a focusing of one's attention externally with little introspection or insight (83). Following this view, in total, 2,501 home-quarantined students from six southwest Chinese universities, it was discovered that participants with probable depression or PTSD experienced more severe alexithymia symptoms (84).

#### Somatization

During the COVID-19 outbreak, it was found that psychosomatic symptoms increased, and changes in perceived threat and biological rhythm, especially intolerance of uncertainty, were influential throughout this increase among 533 participants (85). Likewise, recent research on somatic symptoms associated with COVID-19 among 399 college students and primary school students in February and March 2020 indicates that the prevalence of somatic symptoms in college students was 34.85% (mild, 26.26%; moderate, 8.59%) and in primary school students, the prevalence of somatic symptoms was 2.39% (all mild) (86). The various distortions of the body–mind relationship were recently classified into clusters that corresponded to different interventions, suggesting that in some cases, stress associated with uncertainty heightened several biological disorders with a distinct pathophysiology, while in other cases an increased sensitivity to physical stimuli, along with hyper-reactivity of the autonomic system, forms a “vicious cycle” of learning processes involving biological and psychological dysfunctional mechanisms (e.g., central sensitization, catastrophizing, and selective attention). Another pattern of distortion is demonstrated by the conversion disorder, which indicates the translation of psychological distress into somatic complaints (87).

### 3. The Effect of COVID-19 on High Level Processes

According to MCI, the lack of clarity and ambiguity of individual goals or, on the other hand, radicalization and rigidity manifest a deficiency in high-level processes under uncertainty.

#### Goal Ambiguity

Goal ambiguity occurs with an increased likelihood that no result will be more likely than others. As a result, the individual can no longer confidently ascertain the meaning of any criterion, action or experience. Similarly, the goals or means of coping with the pandemic, vaguely defined by policymakers, cannot narrow the range of potential opportunities. Likewise, uncertainty arises when the resources or costs of coping with the pandemic are not available, or when the number of barriers to achieving reduced rates of contagion is high, so that the system is unable to maintain effective perception and behavioral constraints. This helps to explain the ambiguity caused by COVID-19, which takes place in the form of doubts as to the confidence of decision-makers in obtaining information perceived as true.

#### Goal Conflict

Another source of uncertainty is the conflict between goals, which is the simultaneous activation of competing interpretive mechanisms without particular superiority for a given instance (88) and which may lead to inaction. A significant scope of research on motivation orientation suggests that stagnation and failure require recurrent involvement in chronic assessment of what is the right thing to do rather than engagement in goal directed action (89, 90). Gray and McNaughton (91) argued that goal conflict is one of the precipitators of activation of the behavioral avoidance (or inhibition) system (BIS), reflecting indecision about how best to construct and respond to stimulus (e.g., approach or avoid). If there is clearly no interpretive framework or behavioral response that is most appropriate, there will be parallel activation of many different perceptual and motor response options. Accordingly, response conflicts were also identified as reliably triggering the operation of the anterior cingulate cortex (ACC) and subsequent involvement of the dorsolateral prefrontal cortex (DLPFC) (92–94).

#### Teleological Thinking and Conspiracy Theories

The confusion caused by COVID-19 is expressed in doubts as to the confidence of decision-makers in obtaining information perceived as true and relying on dubious sources of information, such as conspiracy theories, based on the assumption that powerful forces conceal everything. Conspiracy theories are informed by teleological thought, in which everything has a specific secret purpose (95). The use of conspiratorial theories can also be explained on the cognitive level through automated, rapid, and shallow processing of information influenced by limited cognitive resources (96, 97).

#### Complexity and Flexibility versus Rigidity of Goals

Motivated Cue-Integration suggests that trust vs. distrust influences the complexity or rigidity of an individual's narrative. This form of automation takes place in decision-making based on heuristics due to mental shortcuts that reduce the cognitive burden of decision-making, particularly under stress. In fact, excessive rigidity and lack of willingness to explore and confront uncertainty have been linked to a variety of pathological situations, such as obsessive-compulsive disorder (98). These fears are closely linked to the anxiety of existential philosophy that argues that freedom can lead to uncertainty and confusion (99). Such dogmatism can easily spread among individuals and the level of group interpretation and action (100–102). To control the experience of chronic goal-conflict, individuals may engage in goal-shielding, a mechanism that “automatically regulates one's focus by inhibiting potentially distracting alternative objectives” (103). Similarly, radical groups have oversimplified, rigid, and extremist narratives which reduce uncertainty (40).

### Intervention

The intervention's aim is to address self-regulation deficits that manifest via high-level goals (e.g., radicalization, tension, confusion), low-level signals (e.g., low clarity of emotion, somatization), and dissociation or distorted integration of the two. Motivated Cue-Integration suggests that psychological or somatic symptoms associated with emotion dissociation and detachment from genuine psychological needs. Therefore, awareness of the relationship between goals and multisensory data allows for the interpretation of perceptual cues and their potential interaction with psychological needs. The process of motivated cue-integration entails processing the distressing experience by tracking sensations and emotions, identifying the relationships between bodily signals and concepts associated with needs, and developing the ability to examine the scenario from several viewpoints simultaneously. The psychological intervention can be appropriate for both clinical and subclinical conditions where the clinician recognizes low clarity of emotions, dissociation of psychological needs, or cognitive rigidity. The intervention may be combined with other therapeutic intervention or be used as an additional tool like many awareness of sensation techniques (e.g., mindfulness). The intervention begins by identifying a particular area of concern to the patient. The procedure entails paying close attention to emotions as well as interoceptive and exteroceptive cues related to the subject. The perceived sensations are then expressed by associated concepts, which enable movement between perceived sensations and associated words or images. The mentalization of physiological experiences, as well as the association of physical and psychological experiences, can result in a more accurate understanding of the essence of the dilemma or action that can be taken. Paying attention to perceptual cues results in the identification of a specific word or image that carries meaning or reveals an individual's latent personal intent. Tracking bodily sensations linked to concepts or images can result in a broader experience. For example the Focusing technique (104) facilitates the monitoring of sensory perception as well as the association of body signs and verbal ideas that go beyond the linear interpretation that has already been made. By echoing the patient's associations, the therapist facilitates the emergence of unique and fresh information. Exploring the relationship will be handled by personifying the troubling emotion and asking detailed questions about what good meaning is underneath this emotion (e.g., reframing anxiety as need for caution). In the case of people suffering from emotional rigidity or somatization, therapists may be able to help in recognizing the underlying unexpressed desire. The therapist should be aware of any potential biases in perception or ignorance of perceptual cues, allowing for more contemplation and exploration of neglected facets. To increase the clarity of the interaction, this mentalization process should be repeated several times during a single session. Once the patient has experienced relaxation, she documents aspects of the process that she wishes to recall or apply in order to form an integrative image of the experience. The therapist will advise her to write a constructive self-talk statement that summarizes her psychological experience and encourages her to take action.

Because the process of cue-integration is influenced by external information resources, trust has an impact on future prediction and meaning generation. Handling experience and working with a therapist fosters a feeling of secure attachment. Clearly, in acute cases of ambiguity, it is important to reinforce the paths that have contributed to a sense of relative control. This is because adaptive self-regulation fosters trust and comfort zones in an individual's life. Promoting increased awareness of psychological needs, constructive emotion signals, incentive mechanisms, and insight allows for a greater tendency to act.

Taken together, the present paper presented the principles of the theory of MCI (4–6), a novel theory of self-regulation and its applications for the understanding of self-regulation deficiencies under COVID19. According to MCI, individuals create meaning by linking low-level interoceptive and exteroceptive cues and high-level goals, values, and aspirations. Motivated Cue-Integration claims that individuals differ in their associative relationships with their goal system, as well as their interaction with interoceptive and exteroceptive signals and emotions, and how these associations are expressed by words, bodily sensations, mental images, or physical symptoms. The process of MCI is influenced by trust-based relationships with epistemic authority as a trustworthy source of support and information. Psychopathology including deficits in interpreting the social environment are linked to the peculiar form of human interpretation, which creates integration between high level goals and external and low-level internal signals. Psychotherapy is a restructuring of particular and contextual emotional perception for the purpose of self-regulation. Future studies should examine the efficacy of MCI in optimizing MCI as a predictor of adaptive and maladaptive functioning. Such research would advance the state of the art of evidence-based analysis in the areas the creation of meaning, self-regulation, predictive coding, and the potential convergence between these fields.

## Author Contributions

The author confirms being the sole contributor of this work and has approved it for publication.

## Conflict of Interest

The author declares that the research was conducted in the absence of any commercial or financial relationships that could be construed as a potential conflict of interest.
